# Surveillance of Digoxin Concentrations in Critically Ill Individuals with Heart Failure

**DOI:** 10.3390/medicina61081365

**Published:** 2025-07-28

**Authors:** Marek Grochla, Marcin Basiak, Ewa Sztohryn, Anna Szczepańska-Gumulak, Maciej Chylak, Bogusław Okopień, Piotr Knapik

**Affiliations:** 1 Department of Anesthesiology and Intensive Therapy in Zabrze, Medical University of Silesia in Katowice, 40-055 Katowice, Poland; marek.grochla@sum.edu.pl (M.G.);; 2 Department of Internal Medicine and Clinical Pharmacology in Katowice, Medical University of Silesia in Katowice, 40-055 Katowice, Poland; 3 Silesian Centre for Heart Diseases in Zabrze, 41-800 Zabrze, Poland; e.sztohryn@sccs.pl (E.S.); a.szczepanska-gumulak@sccs.pl (A.S.-G.); 4 Centrum Zdrowia Dziecka i Rodziny im. Jana Pawła II w Sosnowcu, 41-218 Sosnowiec, Poland; mchylak@mp.pl

**Keywords:** drug monitoring, intensive care unit, digoxin

## Abstract

*Background and Objectives:* Digoxin is a pharmacological agent of natural origin that is still occasionally administered in the intensive care unit (ICU). The objective of this study was to assess the efficacy of routine therapeutic drug monitoring (TDM) of digoxin in ICU patients with heart failure. *Materials and Methods:* This retrospective, single-center study was conducted using data from the ICU database of the Silesian Center for Heart Diseases in Zabrze, Poland. A total of 980 ICU admissions between January 2018 and July 2023 were screened, and 103 patients met the inclusion criteria. Patients were excluded if they had not received digoxin during hospitalization, had only one digoxin level measurement, or did not meet the established criteria for heart failure. *Results:* Women required significantly lower doses of digoxin compared to men (0.171 ± 0.053 mg vs. 0.224 ± 0.080 mg; *p* < 0.001). Patients who died had significantly higher serum digoxin concentrations than survivors (1.33 ± 0.59 ng/mL vs. 1.03 ± 0.43 ng/mL; *p* = 0.003). Similarly, patients with liver failure had higher digoxin levels compared to those without liver dysfunction (1.31 ± 0.58 ng/mL vs. 1.06 ± 0.46 ng/mL; *p* = 0.016). A weak negative correlation was found between age and the administered dose (r = −0.20; *p* = 0.048), and a weak positive correlation was observed between serum digoxin concentration and NT-proBNP levels (r = 0.23; *p* = 0.048). *Conclusions:* Among ICU patients with multi-organ failure, those with concomitant liver dysfunction tended to reach higher serum digoxin concentrations. Routine therapeutic drug monitoring of digoxin in ICU patients appears beneficial and may help to optimize dosing and reduce adverse effects.

## 1. Introduction

Cardiac glycosides are naturally occurring compounds synthesized by certain plants belonging to the families Apocynaceae, Liliaceae, Ranunculaceae, and Scrophulariaceae. Their name reflects their primary pharmacological effect on cardiac muscle. These compounds are classified into two main groups: cardenolide glycosides—produced by plants such as Digitalis—and bufadienolide glycosides, which are now considered to have limited clinical relevance. The most well-known compound in this group is digoxin, which is derived from Digitalis lanata [1]. The primary mechanism of action of cardiac glycosides involves the inhibition of Na^+^/K^+^-ATPase, an enzyme that regulates the intracellular sodium and potassium balance. Inhibition of this enzyme leads to an increase in intracellular sodium, which in turn promotes the influx of calcium ions via the sodium–calcium exchanger, resulting in enhanced myocardial contractility [1,2]. Cardiac glycosides exhibit a narrow therapeutic index. The human myocardium is particularly sensitive to these agents, with a lethal dose as low as 12 µg/kg, in contrast to 200 µg/kg in mice [1]. Contemporary guidelines place decreasing emphasis on the use of digoxin, primarily due to the evolving pharmacological landscape and the drug’s known toxicity profile. Digoxin exerts a positive inotropic and negative chronotropic effect, which underlies its therapeutic utility [2,3]. However, because of its narrow therapeutic index, therapeutic drug monitoring (TDM) may be warranted to optimize the dosing and minimize complications, particularly in elderly patients, who now constitute the majority of those receiving cardiac glycosides [3]. According to current heart failure (HF) guidelines, digoxin may be considered in selected patients with heart failure with reduced ejection fraction (HFrEF). While some studies have questioned the safety of digoxin in patients with atrial fibrillation (AF), especially regarding long-term outcomes, the guidelines still permit its use in patients with rapid ventricular response and coexisting chronic HF. This approach also applies to cases of acute HF with AF, when the ventricular rate exceeds 110 bpm [2,4]. A 2024 meta-analysis did not include digoxin among the recommended treatments, instead highlighting the increasing use of agents such as ARNIs, SGLT2 inhibitors (flozins), omecamtiv mecarbil, and vericiguat. ARNI and flozins have shown benefit in reducing both the primary endpoints and overall morbidity in HF patients. The updated ESC guidelines introduced redefinitions of HF with mildly reduced ejection fraction (HFmrEF) and HF with preserved ejection fraction (HFpEF). In HFmrEF, diuretics and SGLT2 inhibitors are class I recommendations, whereas ACE inhibitors/ARNI/ARB, mineralocorticoid receptor antagonists (MRAs), and β-blockers have class IIb status. In HFpEF, only diuretics and SGLT2 inhibitors have class I indication. A notable addition to the pharmacological armamentarium is finerenone, which has received a class I recommendation for reducing HF-related outcomes in patients with both diabetes mellitus and chronic kidney disease. Regarding digoxin specifically in HFrEF, the guidelines suggest it may be considered (class IIb) in patients in sinus rhythm who remain symptomatic despite optimized therapy with an ACE inhibitor or ARNI, β-blocker, and MRA. The recommended serum digoxin concentration should remain below 1.2 ng/mL. The DIG trial demonstrated a reduction in HF hospitalizations in HFmrEF patients treated with digoxin, although it did not show a mortality benefit. Conversely, in HFpEF, the DIG-PEF subanalysis did not reveal significant improvements in either mortality or hospitalization rates [4,5,6].

The molecular mechanisms involving Na^+^/K^+^-ATPase and the Na^+^/Ca^2+^ exchanger (NCX) are complex but play a fundamental role in cardiomyocyte function. The sarcoplasmic reticulum (SR) acts as a calcium reservoir, releasing Ca^2+^ during systole and reabsorbing it during diastole. Readers interested in a detailed biochemical explanation may consult the specialized literature [7]. The sodium–potassium pump (Na^+^/K^+^-ATPase) is essential for maintaining electrophysiological homeostasis in the heart. It transports sodium (Na^+^) and potassium (K^+^) ions across the cell membrane against their concentration gradients, using ATP as an energy source. In each cycle, the pump extrudes three Na^+^ ions and imports two K^+^ ions, thereby helping to sustain the resting membrane potential, cell excitability, and secondary active transport of other ions and molecules. When the membrane potential of cardiomyocytes rises above −40 mV, L-type calcium channels (LTCCs)—members of the dihydropyridine receptor (DHP) family—open, allowing an influx of extracellular calcium ions (Ca^2+^). These incoming Ca^2+^ ions trigger the opening of ryanodine receptors (RyR2) on the membrane of the sarcoplasmic reticulum, in a process known as calcium-induced calcium release (CICR). This leads to a further increase in the intracellular calcium concentration, which is critical for myofilament activation and myocardial contraction. RyR2 channels are activated by cytosolic calcium and ATP but inhibited by calmodulin. The reduction in intracellular calcium following contraction is achieved through the concerted action of several calcium transport systems, including Na^+^/K^+^-ATPase (indirectly supporting Ca^2+^ removal), Na^+^/Ca^2+^ exchanger (NCX), and sarcoplasmic/endoplasmic reticulum Ca^2+^-ATPase (SERCA). Cardiac relaxation and cell repolarization depend on the active removal of calcium by SERCA and NCX. Calcium homeostasis in cardiomyocytes is additionally modulated by various systemic and intracellular factors, including the adrenergic nervous system, thyroid hormones, and neurotransmitters [7,8,9,10]. It has been demonstrated that mice and rats exhibit greater susceptibility to cardiac glycosides. This increased sensitivity is attributed to mutations in the α-subunit of Na^+^/K^+^-ATPase. A comparative analysis between ouabain-resistant rats and ouabain-sensitive sheep identified three key amino acid residues in the α-subunit—Gln-111, Asp-121, and Asn-122. Substituting these residues in sheep resulted in a 1000-fold reduction in Na^+^/K^+^-ATPase affinity for cardiac glycosides such as ouabain [11].

Despite evolving treatment strategies, digoxin retains clinical relevance, particularly in the management of supraventricular tachyarrhythmias. It is especially effective in slowing the ventricular rate in patients with atrial fibrillation (AF). Digoxin can be used in combination with β-blockers or non-dihydropyridine calcium channel blockers (e.g., verapamil or diltiazem). In cases of intolerance or contraindication to these agents, digoxin may serve as a therapeutic alternative. It is important to note that co-administration of amiodarone or dronedarone significantly increases serum digoxin concentrations, thereby elevating the risk of toxicity [12].

The aim of this study is to evaluate the clinical utility of routine digoxin therapeutic drug monitoring (TDM) in ICU patients with heart failure, with the goal of optimizing treatment efficacy and minimizing adverse effects.

## 2. Materials and Methods

This was a retrospective-, single-center study conducted at the Intensive Care Unit (ICU) of the Silesian Center for Heart Diseases in Zabrze, Poland. The Center is an academic hospital affiliated with the Medical University of Silesia. The ICU consists of 29 beds: 18 beds are dedicated to post-cardiac surgery patients, 9 are for general ICU admissions, and 2 are located within the cardiology ward.

**Figure 1:**
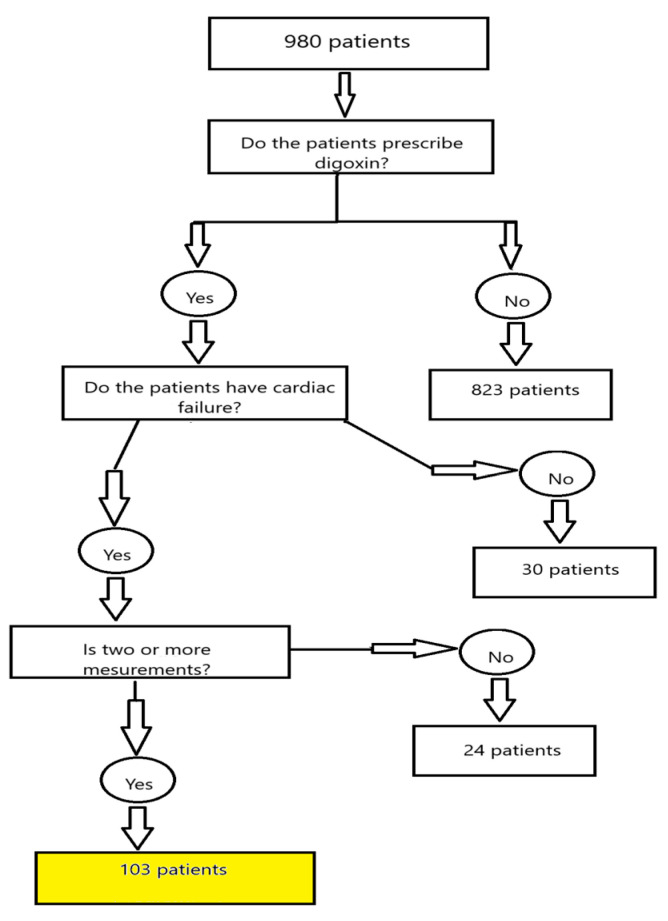
Flow chart explaining the materials and methods.

The present analysis included patients admitted to the general ICU (9 beds) between January 2018 and July 2023 with a diagnosis of heart failure. Out of 980 ICU admissions screened during this period, 103 patients met the inclusion criteria. The remaining 877 admissions were excluded, primarily due to the absence of digoxin treatment, availability of only a single serum digoxin measurement, or failure to meet the predefined diagnostic criteria for heart failure. An explanation of materials and methods is provided in Figure 1.

All data regarding digoxin dosing were extracted from the electronic medical records, and the average daily dosages were calculated accordingly. Serum digoxin concentrations were obtained from the hospital’s therapeutic drug monitoring laboratory, which specializes primarily in immunosuppressive and cardiovascular pharmacotherapy. Quantitative measurements of digoxin levels were performed using the ARCHITECT iDigoxin assay on the ARCHITECT analyzer platform (Abbott Diagnostics, Orlando, FL, USA), a chemiluminescent microparticle immunoassay (CMIA).

Patients were categorized into four groups based on serum digoxin concentrations: below therapeutic: <0.8 ng/mL; therapeutic: 0.8–2.0 ng/mL; above therapeutic: >2.0 ng/mL. Mixed concentrations were values that included both therapeutic and above-therapeutic levels during the ICU stay.

Heart failure was defined as either a previous diagnosis of heart failure, NT-proBNP level > 1000 pg/mL, need for vasopressors or mechanical circulatory support, or reduced left ventricular ejection fraction (LVEF < 45%).

Renal failure was defined as a previously diagnosed chronic kidney disease, the need for renal replacement therapy (RRT), or intravenous diuretic therapy.

Liver failure was defined as a known diagnosis or the presence of clinical/laboratory features, such as an international normalized ratio (INR) >1.5 and total bilirubin levels elevated ≥2-fold above the upper limit of normal.

Pulmonary failure was defined as the need for mechanical ventilation lasting >24 h.

Statistical analyses were performed using Dell Statistica v13 (Round Rock, TX, USA). Descriptive statistics were used to summarize the demographic data. The Kolmogorov–Smirnov test was used to assess the normality of distribution for quantitative variables. Depending on the distribution, comparisons were made using either the two-tailed Student’s *t*-test or the Mann–Whitney U test. Categorical variables were compared using the chi-square test with Yates’ correction. A *p*-value < 0.05 was considered statistically significant.

## 3. Results

A total of 103 patients (10.5%) of all ICU admissions during the study period met the inclusion criteria and were included in the analysis. In as many as 73 patients, serum digoxin concentrations varied substantially during hospitalization, placing them in the “mixed-level” group, defined by the presence of both subtherapeutic and supratherapeutic values outside the 0.8–2.0 ng/mL therapeutic range. The distribution of patients by serum digoxin level categories is presented in Table 1.

Female patients required significantly lower doses of digoxin compared to males (0.171 ± 0.053 mg vs. 0.224 ± 0.080 mg, *p* < 0.001).

Patients who died during hospitalization had significantly higher mean digoxin concentrations compared to survivors (1.33 ± 0.59 ng/mL vs. 1.03 ± 0.43 ng/mL, *p* = 0.003). The overall mortality rate in the study population was 42.7%. Additionally, patients with liver failure had significantly higher digoxin concentrations than those without (1.31 ± 0.58 ng/mL vs. 1.06 ± 0.46 ng/mL, *p* = 0.016). Further comparisons are presented in Table 2.

**Figure 2:**
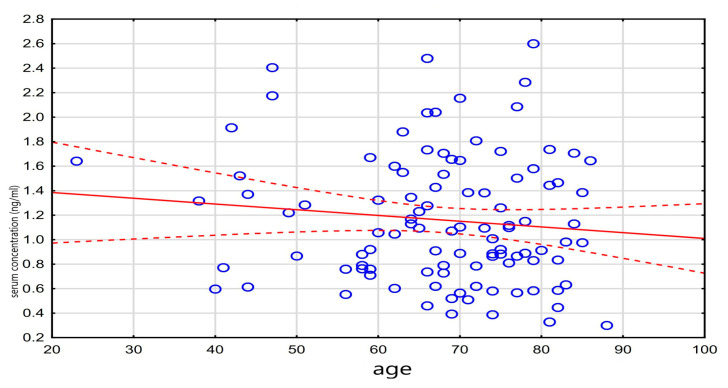
Relationship between death and drug level.

Among the patients who died, liver failure was significantly more common (56.1% vs. 43.9%, *p* = 0.042). These patients also more frequently required epinephrine (52.3% vs. 47.7%, *p* = 0.018) and vasopressin (65% vs. 35%, *p* = 0.046). The relationship between digoxin concentration and mortality is illustrated in Figure 2.

**Figure 3:**
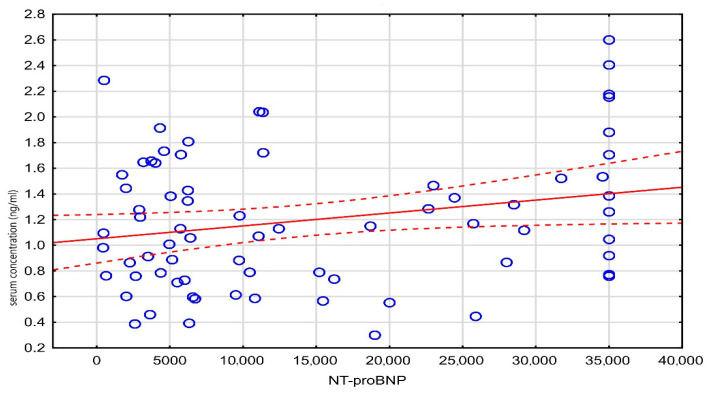
Scatter plot of serum concentration against NT-proBNP.

A weak, statistically significant negative correlation was observed between age and the administered digoxin dose (*r* = −0.20, *p* = 0.048), while a weak positive correlation was found between the digoxin concentration and NT-proBNP levels (*r* = 0.23, *p* = 0.048). These results are detailed in Table 3, and the correlation between the digoxin level and NT-proBNP is visualized in Figure 3.

In the multivariate analysis, liver failure remained a statistically significant predictor of elevated digoxin concentrations in both the full model (*p* = 0.016) and the reduced model (*p* = 0.002). The full multivariate results are shown in Table 4.

## 4. Discussion

Digoxin is one of the oldest pharmacological agents still in clinical use, with a history spanning over 200 years [13]. However, with the advent of novel therapies and technologies for the treatment of heart failure, its clinical relevance has significantly diminished. According to the 2021 heart failure guidelines, digoxin carries a Class IIb recommendation (may be considered) in symptomatic patients despite optimal treatment with the so-called “big four” drug classes: ACE inhibitors (ACEIs) or angiotensin II receptor blockers (ARBs), SGLT2 inhibitors (flozins), mineralocorticoid receptor antagonists (MRAs), and β-blockers [4]. In the 2023 update to these guidelines, no new evidence or recommendations regarding digoxin were introduced. Importantly, digoxin has a relatively long half-life of approximately 36 h, which contributes to its increased risk of drug accumulation and interactions, especially in elderly patients and those with impaired renal function [2]. The primary organ responsible for digoxin elimination is the kidney, while only approximately 30% of the drug is metabolized by the liver [14]. In patients with heart failure, renal function is frequently impaired due to low cardiac output, leading to secondary renal dysfunction. In the present study, over 95% of patients met the criteria for renal failure, which may explain the lack of statistically significant differences in digoxin serum concentrations between patients with and without renal impairment. A similar trend was observed in patients receiving renal replacement therapy (RRT), where no significant association with digoxin levels was found. This finding may be attributed to two possible factors: insufficient statistical power due to small sample sizes in subgroup analyses, or heterogeneity resulting from the combined classification of different RRT modalities (e.g., intermittent hemodialysis, continuous veno-venous hemofiltration). Currently, digoxin is not a major focus of clinical research, and as such, a literature search in the PubMed database revealed no recent publications specifically evaluating the effect of RRT on serum digoxin concentrations. However, data from older and secondary sources suggest that RRT is not effective in removing digoxin from circulation, largely due to its large volume of distribution and high tissue binding affinity [14,15]. A related agent currently under clinical investigation is digitoxin, another cardiac glycoside derived from the Digitalis plant group. Unlike digoxin, digitoxin is primarily metabolized in the liver, with minimal renal excretion. This pharmacokinetic profile is potentially advantageous, as secondary hepatic dysfunction in the setting of heart failure (HF) is less common than renal impairment [4]. The ongoing Digit-HF trial, conducted in Germany, aims to evaluate whether digitoxin can improve clinical outcomes in patients with heart failure. In 2023, preliminary findings from the study were published, focusing primarily on dosing recommendations [16]. The final results are still pending, and further data from the German research group are anticipated to clarify the therapeutic role and safety profile of digitoxin in modern heart failure management. There is evidence in the literature suggesting that higher serum digoxin concentrations are associated with increased mortality [17]. This observation was also confirmed in the present study. Although patients who died had digoxin levels within the therapeutic range, a positive correlation between higher digoxin concentrations and mortality was observed. These findings support the recommendation that in critically ill ICU patients, digoxin levels should be maintained at the lower end of the therapeutic range while still ensuring clinical efficacy. Nonetheless, the role of digoxin in overall mortality remains controversial. While some studies have shown that digoxin therapy is associated with increased all-cause or cardiovascular mortality [17,18,19,20,21,22,23,24,25], other reports have presented conflicting results or failed to confirm this association. Additionally, certain studies have suggested that digoxin may reduce hospital readmission rates, particularly in patients with chronic heart failure [13,17,19,20,21,23], although this benefit is not consistently reported across all investigations. On the other hand, concerns have been raised regarding the pro-arrhythmic potential of digoxin. Several publications have indicated an increased risk of ventricular tachycardia or ventricular fibrillation (VT/VF) associated with its use, especially in patients with structural heart disease or electrolyte disturbances [26]. As previously noted, approximately 30% of digoxin is metabolized by the liver, while the majority is excreted unchanged by the kidneys [2]. In the presence of renal failure, hepatic metabolism may play a compensatory role in digoxin clearance. However, a significant clinical challenge arises when heart and kidney failure are accompanied by hepatic dysfunction, as this combination may severely impair drug elimination. In this study, over 95% of patients exhibited signs of renal failure, while approximately 40% had laboratory or clinical signs of liver failure. Among patients receiving comparable doses of digoxin, those with liver failure demonstrated significantly higher serum concentrations than patients without hepatic involvement. These findings suggest a possible increased digoxin retention in patients with multi-organ failure (MOF). However, further research is needed to confirm whether MOF contributes to clinically significant digoxin accumulation and toxicity. In addition to organ dysfunction, drug–drug interactions also play a key role in modulating digoxin levels. These interactions can be either pharmacodynamic or pharmacokinetic. A classic pharmacodynamic interaction involves loop or thiazide diuretics, which lower serum potassium levels and thereby enhance digoxin’s myocardial effects and toxicity risk. Pharmacokinetic interactions include inhibition of P-glycoprotein (P-gp) and cytochrome P450 3A4 (CYP3A4), both of which are involved in digoxin transport and metabolism. For example, amiodarone is a potent P-gp inhibitor, leading to reduced digoxin clearance and elevated plasma levels. Calcium channel blockers (such as verapamil or diltiazem) inhibit both P-gp and CYP3A4, thereby also increasing digoxin exposure [2,27]. These factors highlight the need for individualized therapeutic drug monitoring (TDM) in ICU patients, especially in those with organ dysfunction or on polypharmacotherapy. The observed mortality rate in this study was 42.7%, which is comparable to rates reported in other Polish ICU cohorts of patients with advanced heart failure [28,29,30].

This study has several limitations. First, it was conducted as a retrospective, single-center analysis, which inherently limits both the generalizability and the granularity of the findings. The retrospective design also precluded randomized patient selection and prospective follow-up, which may introduce selection bias and limit the ability to establish causality. Another limitation is the relatively small sample size, which may have reduced statistical power, especially in subgroup analyses (e.g., RRT modalities or hepatic dysfunction). Additionally, digoxin was administered infrequently and sometimes only for short-term rate control in atrial fibrillation, which hindered accurate assessment of daily maintenance dosing in certain cases. On the other hand, a strength of this study is its setting in a highly specialized academic center, which provides care primarily for patients with advanced cardiovascular diseases, particularly heart failure—a condition often referred to as the “epidemic of the 21st century.” This ensures that the study cohort is clinically relevant and well-characterized.

## 5. Conclusions

In critically ill patients with multi-organ failure (MOF) admitted to the ICU, serum digoxin concentrations are significantly higher in those with concomitant liver dysfunction. These findings support the value of routine therapeutic drug monitoring (TDM) of digoxin in ICU patients, as it may aid in optimizing therapy and reducing the risk of toxicity.

## Figures and Tables

**Table 1:** Comparison between digoxin level and demographic parameters.

	Therapeutic Level			Mixed Level			Above Terap. Level			Below Terap. Level			*p*	*p*	*p*	*p*	*p*	*p*
	N	Mean	SD	N	Mean	SD	N	Mean	SD	N	Mean	SD	1 vs. 2	1 vs. 3	1 vs. 4	2 vs. 3	2 vs. 4	3 vs. 4
Digit. serum Con.(ng/mL)	15	1.390	0.184	73	1.203	0.501	2	2.283	0.279	13	0.488	0.109	0.132	**0.008**	**0.000**	**0.001**	**0.000**	**0.000**
Digoxin dose (mg)	15	0.217	0.097	73	0.203	0.070	2	0.160	0.042	13	0.222	0.093	0.507	0.325	0.865	0.439	0.402	0.289
Age (year)	15	70.33	13.72	73	66.33	12.14	2	71.50	7.78	13	72.77	11.68	0.253	0.900	0.602	0.558	0.085	0.892
Weight (kg)	15	82.53	17.14	70	90.53	28.61	2	92.00	11.31	13	85.77	20.85	0.197	0.562	0.694	0.925	0.468	0.705
ICU stay (day)	15	23.93	20.85	73	28.41	18.74	2	17.00	4.24	13	22.00	11.13	0.388	0.614	0.780	0.384	0.245	0.719
LVEF (%)	14	35.14	17.50	69	31.78	15.84	2	55.00	0.00	11	30.64	15.58	0.423	0.069	0.435	**0.026**	0.805	**0.029**
RVSP (mmHg)	10	34.70	19.12	39	40.44	22.17	1	70.00	49.50	4	45.25	22.70	0.187	**0.008**	0.146	**0.019**	0.452	0.073
NT-proBNP (pg/mL)	11	16,020	13,907	53	14,615	12,643	0			8	11,282	8480	0.736		0.419		0.486	
	N	*n*	%	N	*n*	%	N	*n*	%	N	*n*	%						
Women	15	10	66.7%	73	21	28.8%	2	2	100.0%	13	1	7.7%	**0.012**	0.884	**0.005**	0.168	0.208	**0.037**
Death	15	7	46.7%	73	30	41.1%	2	2	100.0%	13	5	38.5%	0.912	0.506	0.956	0.349	0.898	0.388
SCD	15	2	13.3%	73	25	34.2%	2	1	50.0%	13	5	38.5%	0.196	0.772	0.274	0.771	0.982	0.642
AF	15	15	100.0%	73	66	90.4%	2	2	100.0%	13	11	84.6%	0.468	-	0.400	0.440	0.891	0.602
Liver failure	15	4	26.7%	73	33	45.2%	2	1	50.0%	13	3	23.1%	0.299	0.884	0.827	0.558	0.236	0.954
Kidney failure	15	15	100.0%	73	68	93.2%	2	2	100.0%	13	13	100.0%	0.666	-	-	0.292	0.742	-
Respiratory failure	15	15	100.0%	73	68	93.2%	2	2	100.0%	13	12	92.3%	0.666	-	0.942	0.292	0.631	0.264
CRRT	15	9	60.0%	73	50	68.5%	2	2	100.0%	13	8	61.5%	0.737	0.746	0.761	0.860	0.864	0.788
Dobutamine	15	7	46.7%	73	51	69.9%	2	1	50.0%	13	7	53.8%	0.154	0.506	1.000	0.860	0.416	0.509
Norepinephrine	15	15	100.0%	73	67	91.8%	2	2	100.0%	13	10	76.9%	0.557	-	0.175	0.369	0.262	0.849
Epinephrine	15	8	53.3%	73	50	68.5%	2	2	100.0%	13	5	38.5%	0.407	0.621	0.684	0.860	0.078	0.388
Milrinone	15	6	40.0%	73	15	20.5%	2	1	50.0%	13	1	7.7%	0.202	0.621	0.126	0.898	0.477	0.602
Vasopressin	15	1	6.7%	73	14	19.2%	2	2	100.0%	13	3	23.1%	0.426	**0.024**	0.486	0.060	0.958	0.179
Levosimendan	15	1	6.7%	73	6	8.2%	2	0	0.0%	13	1	7.7%	0.748	0.221	0.528	0.369	0.627	0.264
IABP	15	2	13.3%	73	20	27.4%	2	0	0.0%	13	2	15.4%	0.413	0.536	0.699	0.957	0.569	0.602
LVAD	15	0	0.0%	73	5	6.8%	2	0	0.0%	13	0	0.0%	0.666	-	-	0.292	0.742	-
ECMO	15	2	13.3%	73	7	9.6%	2	0	0.0%	13	1	7.7%	0.975	0.536	0.896	0.440	0.763	0.264
NO	15	2	13.3%	73	12	16.4%	2	1	50.0%	13	1	7.7%	0.930	0.772	0.896	0.772	0.696	0.602
Readmission to ICU	15	1	6.7%	73	13	17.8%	2	0	0.0%	13	2	15.4%	0.492	0.221	0.896	0.772	0.854	0.602

ICU: intensive care unit; SCD: sudden cardiac death; AF: atrial fibrillation; CRRT: continuous renal replacement therapy; IABP: intra-aortic balloon pump; LVAD: left ventricular assist device; ECMO: extracorporeal membrane oxygenation; NO: nitric oxide; LVEF: left ventricular ejection fraction; RVSP: right ventricular systolic pressure; NT-proBNP: natriuretic peptide tests.

**Table 2:** Comparison between digoxin and dose regarding demographics or status.

	Yes			No			*p*	
	*n*	Mean	SD	*n*	Mean	SD		
Concentration (ng/mL)	34	1.25	0.47	69	1.12	0.55	0.249	Women
Digoxin dose (mg)	34	0.171	0.053	69	0.224	0.080	**0.000**	
Concentration (ng/mL)	44	1.33	0.59	59	1.03	0.43	**0.003**	Death
Digoxin dose (mg)	44	0.207	0.096	59	0.206	0.058	0.976	
Concentration (ng/mL)	33	1.05	0.49	70	1.21	0.54	0.155	SCD
Digoxin dose (mg)	33	0.205	0.075	70	0.207	0.078	0.921	
Concentration (ng/mL)	41	1.31	0.58	62	1.06	0.46	**0.016**	Liver failure
Digoxin dose (mg)	41	0.214	0.075	62	0.202	0.077	0.415	
Concentration (ng/mL)	13	0.49	0.11	90	1.26	0.49	**0.000**	Below therapeutic level
Digoxin dose (mg)	13	0.222	0.093	90	0.204	0.074	0.430	
Concentration (ng/mL)	15	1.39	0.18	88	1.12	0.55	0.067	Therapeutic level
Digoxin dose (mg)	15	0.217	0.097	88	0.205	0.073	0.557	
Concentration (ng/mL)	2	2.28	0.28	101	1.14	0.50	**0.002**	Above therapeutic level
Digoxin dose (mg)	2	0.160	0.042	101	0.207	0.077	0.387	
Concentration (ng/mL)	73	1.20	0.50	30	1.06	0.57	0.207	Mixed level
Digoxin dose (mg)	73	0.203	0.070	30	0.216	0.092	0.441	
Concentration (ng/mL)	69	1.20	0.54	34	1.07	0.48	0.245	CRRT
Digoxin dose (mg)	69	0.203	0.083	34	0.214	0.062	0.484	
Concentration (ng/mL)	10	1.01	0.34	93	1.18	0.54	0.335	ECMO
Digoxin dose (mg)	10	0.218	0.063	93	0.205	0.078	0.621	
Concentration (ng/mL)	16	1.23	0.52	87	1.15	0.53	0.568	Readmission to ICU
Digoxin dose (mg)	16	0.196	0.063	87	0.208	0.079	0.560	

ICU: intensive care unit; SCD: sudden cardiac death; CRRT: continuous renal replacement therapy; ECMO: extracorporeal membrane oxygenation.

**Table 3:** Correlation between parameters.

		Age	Weight	ICU Days Hospitalization	Digit. Serum Concentr. (ng/mL)	Digitalis Dose (mg)	NT-ProBNP
Age	r		−0.21	−0.10	−0.11	−0.20	−0.18
	*n*		100	103	103	103	72
	*p*		0.037	0.300	0.269	0.048	0.129
Weight	r	−0.21		−0.12	-0.03	0.16	−0.20
	*n*	100		100	100	100	69
	*p*	0.037		0.220	0.760	0.111	0.098
ICU days hospitalization	r	−0.10	−0.12		−0.05	-0.11	−0.19
	*n*	103	100		103	103	72
	*p*	0.300	0.220		0.594	0.253	0.106
Digit. serum concentr. (ng/mL)	r	−0.11	−0.03	−0.05		0.04	0.23
	*n*	103	100	103		103	72
	*p*	0.269	0.760	0.594		0.672	0.048
Digitalis dose (mg)	r	−0.20	0.16	−0.11	0.04		−0.03
	*n*	103	100	103	103		72
	*p*	0.048	0.111	0.253	0.672		0.801
NT-proBNP	r	−0.18	−0.20	-0.19	0.23	−0.03	
	*n*	72	69	72	72	72	
	*p*	0.129	0.098	0.106	0.048	0.801	

ICU: intensive care unit; NT-proBNP: natriuretic peptide tests.

**Table 4:** Results of the multivariate analysis of digoxin level and dependent variables.

Full Model			
N = 93	coefficient	SD	*p*
Intercept	0.8797	0.6178	0.1588
Age (year)	−0.0037	0.0055	0.5047
Weight (kg)	−0.0027	0.0028	0.3537
LVEF (%)	0.0066	0.0051	0.1992
Digoxin dose (mg)	1.0157	0.8243	0.2220
Women	0.1898	0.1351	0.1644
SCD	−0.1456	0.1284	0.2607
AF	−0.0536	0.2125	0.8016
Liver failure	0.3189	0.1290	0.0158
Kidney failure	0.1001	0.2955	0.7357
Respiratory failure	0.0427	0.2354	0.8567
CRRT	−0.0547	0.1327	0.6815
Dobutamine	−0.0657	0.1307	0.6168
Norepinephrine	0.1412	0.2092	0.5021
Epinephrine	−0.0546	0.1312	0.6786
Milrinone	0.1317	0.1639	0.4245
Vasopressin	0.1051	0.1572	0.5060
Levosimendan	0.1233	0.2506	0.6241
IABP	0.2352	0.1631	0.1538
LVAD	−0.0623	0.2813	0.8255
ECMO	−0.5607	0.2313	0.0179
**Reduced Model**			
N = 93	coefficient	SD	*p*
Intercept	0.9407	0.0812	0.0000
Liver failure	0.3434	0.1085	0.0021
Women	0.2201	0.1072	0.0431
ECMO	−0.4397	0.1803	0.0167
NO	0.2774	0.1506	0.0689

SCD: sudden cardiac death; AF: atrial fibrillation; CRRT: continuous renal replacement therapy; IABP: intra-aortic balloon pump; LVAD: left ventricular assist device; ECMO: extracorporeal membrane oxygenation; NO: nitric oxide; LVEF: left ventricular ejection fraction.

## Data Availability

The data are contained within this article.

## Abbreviations

The following abbreviations are used in this manuscript:

ACEI	Angiotensin-converting-enzyme inhibitors
ARB	Angiotensin II receptor blocker
AF	Atrial fibrillation
ß-blocker	Beta blocker
Ca-blocker	Calcium blocker
CRRT	Continuous renal replacement therapy
CYP3A4	Cytochrome P450 3A4
ECMO	Extracorporeal membrane oxygenation
IABP	Intra-aortic balloon pump
ICU	Intensive care unit
HF	Heart failure
HFrEF	Heart failure with reduced ejection fraction
LVEF	Left ventricular ejection fraction
LVAD	Left ventricular assist device
MOF	Multi-organ failure
MRA	Mineralocorticoid receptor antagonist
Na, K-ATPase	Sodium–potassium ATP pump
NO	Nitric oxide
NT-proBNP	Natriuretic peptide tests
SCD	Sudden cardiac death
RRT	Renal replacement therapy
RVSP	Right ventricular systolic pressure
VF	Ventricular fibrillation
VT	Ventricular tachycardia

## References

[1] Kołodziejczyk A. (2006). Glikozydy nasercowe. Naturalne Związki Organiczne.

[2] Ritter J., Flower R., Mirowska-Guzel D., Okopień B. (2021). Rang and Dale Farmakologia.

[3] Charfi R., Sassi M.B. (2020). Digoxin therapeutic drug monitoring: Age influence and adverse events. Tunis. Med..

[4] Mc Donagh T.A., Metra M. (2021). 2021 ESC Guidelines for the diagnosis and treatment of acute and chronic heart failure: Developed by the Task Force for the diagnosis and treatment of acute and chronic heart failure of the European Society of Cardiology (ESC) with the special contribution of the Heart Failure Association (HFA) of the ESC. Eur. Heart J..

[5] Lavalle C., Mariani M.V. (2024). Efficacy of Modern Therapies for Heart Failure with Reduced Ejection Fraction in Specific Population Subgroups: A Systematic Review and Network Meta-Analysis. Cardiorenal Med..

[6] Mc Donagh T.A., Metra M. (2023). 2023 Focused Update of the 2021 ESC Guidelines for the diagnosis and treatment of acute and chronic heart failure: Developed by the Task Force for the diagnosis and treatment of acute and chronic heart failure of the European Society of Cardiology (ESC) with the special contribution of the Heart Failure Association (HFA) of the ESC. Eur. Heart J..

[7] Sutanto H., Lyon A. (2020). Cardiomyocyte calcium handling in health and disease: Insights from in vitro and in silico studies. J. Prog. Biophys. Mol. Biol..

[8] Brzozowski T. (2019). Konturek. Fizjologia Człowieka.

[9] Pagel P.S., Freed J.K. (2017). Cardiac Physiology. Kaplans’s Cardiac Anesthesia for Cardiac and Noncardiac Surgery.

[10] Kieć-Wilk B., Petkow-Dimitrow P. (2009). Role of impaired calcium homeostasis in the development of cardiac hypertrophy. Kardiol. Pol..

[11] O’Brien W.J., Wallick E.T. (1993). Amino Acid residues of the Na,K-ATPase involved in oubain sensititivity do not bind the sugar moiety of cardiac glycosides. J. Biol. Chem..

[12] Merino J.L., Tamargo J. (2025). Practical Compendium of Antiarrhytmic Drugs: A Clinical Consensus Statement of the European Heart Rhythm. Association of the ESC. Europace.

[13] Alkhawam H., Abo-salem E. (2019). Effect of digitalis level on readmission and mortality rate among heart failure reduced ejection fraction patients. Heart Lung.

[14] Brunton L.L., Lazo J.S., Buczko W., Krzemiński T.F. (2007). Farmakologia Goodmana & Gilmana.

[15] Urząd Rejestracji Produktów Leczniczych, Wyrobów Medycznych i Produktów Biobójczych. https://leki.urpl.gov.pl/files/43_Digoxin_WZF_tabl_250mcg.pdf.

[16] Bavendiek U., Großhennig A. (2023). Simple and safe digitoxin dosing in heart failure based on data from the DIGITHF trial. Clin. Res. Cardiol..

[17] Adams K.F., Patterson J.H. (2005). Relationship of Serum Digoxin Concentration to Mortality and Morbidity in Women in the Digitalis Investigation Group Trial. J. Am. Coll. Cardiol..

[18] Washam J.B., Stevens S.R. (2015). Digoxin use in patients with atrial fibrillation and adverse cardiovascular outcomes: A retrospective analysis of the Rivaroxaban Once Daily Oral Direct Factor Xa Inhibition Compared with Vitamin K Antagonism for Prevention of Stroke and Embolism Trial in Atrial Fibrillation (ROCKET AF). Lancet.

[19] Lam P.H., Packer M. (2020). Digoxin Initiation and Outcomes in Patients with Heart Failure with Preserved Ejection Fraction. Am. J. Med..

[20] Singh S., Moore H. (2020). Digoxin Initiation and Outcomes in Patients with Heart Failure (HFrEF and HFpEF) and Atrial Fibrillation. Am. J. Med..

[21] Al-khateeb M., Qureshi W.T. (2017). The impact of digoxin on mortality in patients with chronic systolic heart failure: A propensity-matched cohort study. Int. J. Cardiol..

[22] Wu S., Yang Y. (2017). Predictors of digoxin use and risk of mortality in ED patients with atrial fibrillation. Am. J. Emerg. Med..

[23] Llàcera P., Núñezb J. (2019). Digoxin and prognosis of heart failure in older patients with preserved ejection fraction: Importance of heart rate. Results from an observational and multicenter study. Eur. J. Intern. Med..

[24] Freeman J.V., Reynolds K. (2015). Digoxin and Risk of Death in Adults with Atrial Fibrillation The ATRIA-CVRN Study. Circ. Arrhythm. Electrophysiol..

[25] Mulder B.A., Van Veldhuisen D.J. (2014). Digoxin in patients with permanent atrial fibrillation: Data from the RACEII study. Heart Rhythm.

[26] Lee A.Y., Kutyifa V. (2015). Digoxin therapy and associated clinical outcomes in the MADIT-CRT trial. Heart Rhythm.

[27] Tuszyński P.K. (2024). Uman-Ntuk E.Interakcje leków układu krążenia. Istotne Interakcje Leków Praktyczny Przewodnik.

[28] Grochla M., Saucha W. (2020). Readmissions to General ICUs in a Geographic Area of Poland Are Seemingly Associated with Better Outcomes. Int. J. Environ. Res. Public Health.

[29] Weigl W., Adamski J. (2018). ICU mortality and variables associated with ICU survival in Poland: A nationwide database study. Eur. J. Anaesthesiol..

[30] Adamski J., Goraj R. (2015). The differences between two selected intensive care units incentral and northern Europe—Preliminary observation. Anaesthesiol. Intensive Ther..

